# Functionalized micro-capillary film for the rapid at-line analysis of IgG aggregates in a cell culture bioreactor

**DOI:** 10.1080/19420862.2015.1065365

**Published:** 2015-07-15

**Authors:** Matthew J Townsend, David E Gruber, Marcel Kuiper, Radu A Lazar, Ray P Field, Richard E Turner, Nigel KH Slater

**Affiliations:** 1 Department of Chemical Engineering and Biotechnology; University of Cambridge; New Museums Site; Cambridge, UK; 2 MedImmune; Granta Park; Cambridge, UK

**Keywords:** microcapillary film, chromatography, antibody, aggregate, monitoring, CHO

## Abstract

A micro-capillary film has been developed that offers the potential for an at-line analytical tool for rapid aggregate analysis during biopharmaceutical antibody production. A non-porous walled micro-capillary film (NMCF) with cation exchange functionality was demonstrated to act as a chromatography medium that could be operated with high linear fluid velocities and was highly resistant to blockage by entrained particulates, including cells. The NMCF containing 19 parallel microcapillaries was prepared using a melt extrusion process from poly(ethylene-vinyl alcohol) copolymer (EVOH). The NMCF-EVOH was modified to have cation-exchange functionality (NMCF-EVOH-SP) and shown to differentially bind monomer and aggregated species of IgG antibody directly from a bioreactor. The use of NMCF-EVOH-SP to quantify aggregate concentrations in monoclonal antibody preparations in less than 20 minutes was demonstrated.

## Abbreviations

CHO: Chinese hamster ovary
CM: conditioned medium
CV: column volumes
EVOH: poly(ethylene-vinyl alcohol) copolymer
HCP: host cell proteins
IgG: immunoglobulin G
mAb: monoclonal antibody
MES: 2-(*N*-morpholino)ethanesulfonic acid hydrate
NMCF: non-porous walled micro-capillary film
PBS: phosphate-buffered saline
SEC HPLC: size-exclusion chromatography high performance liquid chromatography

## 

Therapeutic mAbs are ideally suited for the treatment of a wide variety of diseases due to their high specificity for disease-related antigens, and over 40 have been approved for clinical use.^1^ They can also serve as targeting molecules for the delivery of drugs and toxins to specific cells.^2^ However, mAbs are expensive to manufacture, complex to purify and require careful monitoring during production to ensure that they are fit for clinical use. Quality control testing of mAb preparations includes assays for the levels of product purity, as well as undesirable product variants such as aggregates.^3-6^

IgG aggregation can occur throughout the production process,^7^ with reported levels reaching as high as 30% within a bioreactor.^8^ Whereas visible aggregates tend to be insoluble and are easily removed with filtration before downstream purification,^9^ smaller, soluble aggregates are more difficult to remove. The ability to measure the accumulation of aggregates, at-line, during fermentation and throughout purification would be beneficial to bioprocessing. Real-time, or near real-time, at-line aggregation analysis would represent an important step toward dynamic process control strategies could be made.

Several methodologies for aggregate analysis exist. The industry's standard method for the measurement of aggregation, size-exclusion chromatography high performance liquid chromatography (SEC HPLC), requires substantial sample pre-treatment, including both cell removal and purification (e.g., Protein A affinity chromatography) prior to analysis. SEC HPLC analysis of multiple samples typically takes several hours to complete, and can only be regarded as an offline measurement. It is therefore not an appropriate tool for bioreactor process control.

A possible alternative technology is non-porous walled micro-capillary film (NMCFs), which is composed of micro-structured continuous micro-capillaries that can be extruded from a range of thermoplastic polymers in a low cost, scalable procedure.^10,11^ The material offers an attractive advantage for in-process parameter control as a non-porous substrate because the film can accommodate crude cell-containing samples without pre-treatment. Film mass transfer resistance is low compared to conventional adsorbents, such as packed bed resins, and higher liquid superficial flow rates are achievable with NMCF, producing sharp chromatography peaks. Previously, Darton et al.^12^ demonstrated the use of NMCF-EVOH-SP as a cation exchange adsorbent capable of binding and separating cationic proteins (lysozyme, cytochrome C) and the anion-exchange NMCF purification of lentivirus in the presence of host cells.^13^

IgG antibodies are one of the most charged proteins present in serum-free bioreactor harvest at neutral pH.^14^ Based on this distinctive feature, cation exchange chromatography has been described in several studies as a low-cost, alternative first capture step to Protein A affinity chromatography.^15-17^ We hypothesized that the cation exchange surface of the NMCF-EVOH-SP should be able to isolate IgG antibody in the presence of cells and host cell proteins (HCP).

Here, we demonstrate that a chromatography method based on cation-exchange NMCF can indeed be used to detect and quantify aggregated antibody in both purified and unpurified samples. The ability of the analytical test to discriminate and quantify aggregates in the presence of complex sample matrices, including HCP and other cell culture media components was determined.

**Figure 1.**
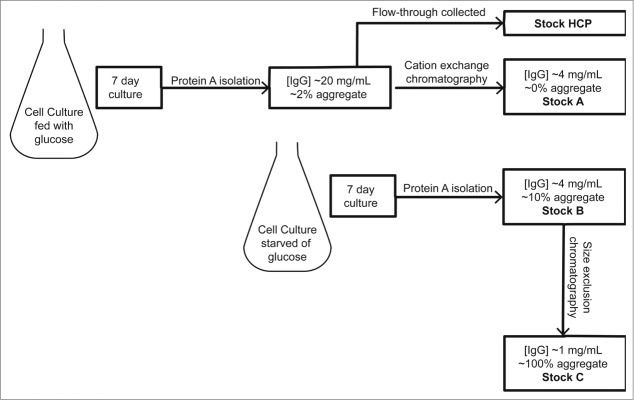
Diagram summarizing the creation of the 3 Stocks A, B and C used to blend IgG samples with varying aggregate and HCP levels employed within this study.

**Figure 2.**
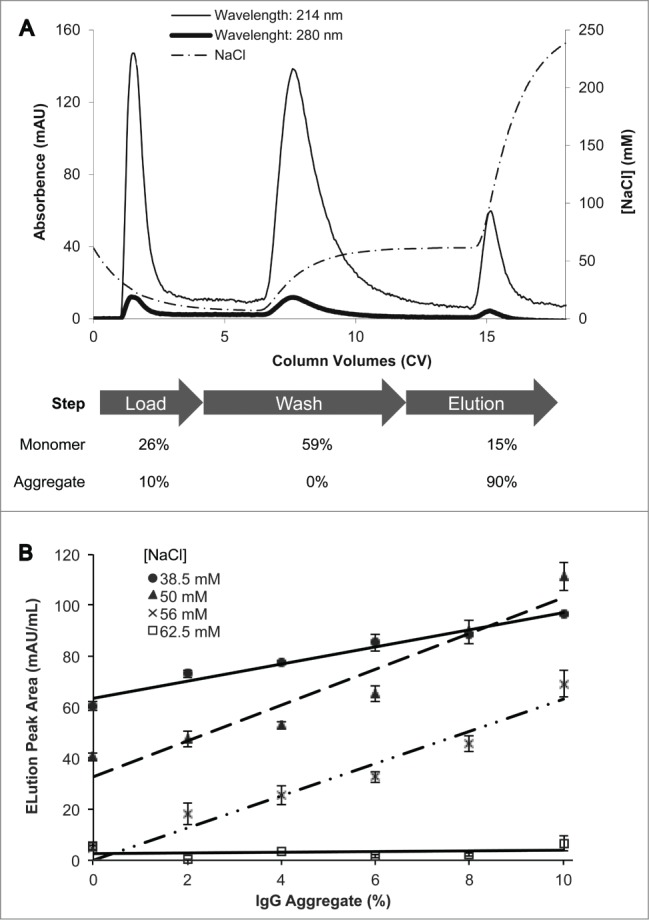
(**A**) Binding of IgG monomer and aggregate to NMCF-EVOH-SP. The chromatogram shows absorbance at 214 and 280 nm as well as [NaCl] against CV for a 100 μL Stock B (10% aggregate) sample at 1 mg/mL. The breakthrough, wash and elution fractions were analyzed with SEC HPLC to determine the relative percentage of IgG monomer and aggregate. (**B**) Elution peak area plotted against percentage of sample aggregate (0%, 2%, 4%, 6%, 8%, and 10%) at a fixed concentration of 1 mg/mL IgG antibody. Four wash conditions were tested (38.5 mM, 50 mM, 56 mM, and 62.5 mM NaCl) and were performed in triplicate. The error bars are the standard error of the mean; 38.5 mM, 50 mM, and 56 mM all have R^2^ values above 0.9 with a linear regression.

The following section describes our experimental results. In order to establish whether NMCF-EVOH-SP is capable of differentially binding monomeric and aggregated IgG, a Stock B sample (**Fig. 1**) containing 10% aggregated and 90% monomeric IgG was loaded, and then followed by a wash step using buffer containing 50 mM NaCl and finally an elution step with buffer containing 250 mM NaCl. The results show that the breakthrough and wash peaks combined contain the majority of the monomeric IgG (85%), whereas the elution peak contains the majority of aggregated IgG (90%). (**Fig. 2A**). This demonstrates that aggregated and monomeric IgG bind differentially to NMCF-EVOH-SP. In addition, the peak amplitude is higher at a wavelength of 214 nm compared to 280 nm; therefore, detection at 214 nm was chosen for subsequent experiments. Different NaCl concentrations (38.5 – 62.5 mM) were tested to optimize elution peak area. This was tested with a blend of 2 IgG stocks used to create a range of aggregate IgG from 0% – 10%. **Figure 2B** shows that a linear increase in elution peak area was obtained with 38.5, 50 and 56 mM NaCl wash buffers, but no increase in elution peak area was observed when a 62.5 mM NaCl wash buffer was used. Subsequent experiments used 50 mM as the NaCl concentration for the wash buffer.

**Figure 3.**
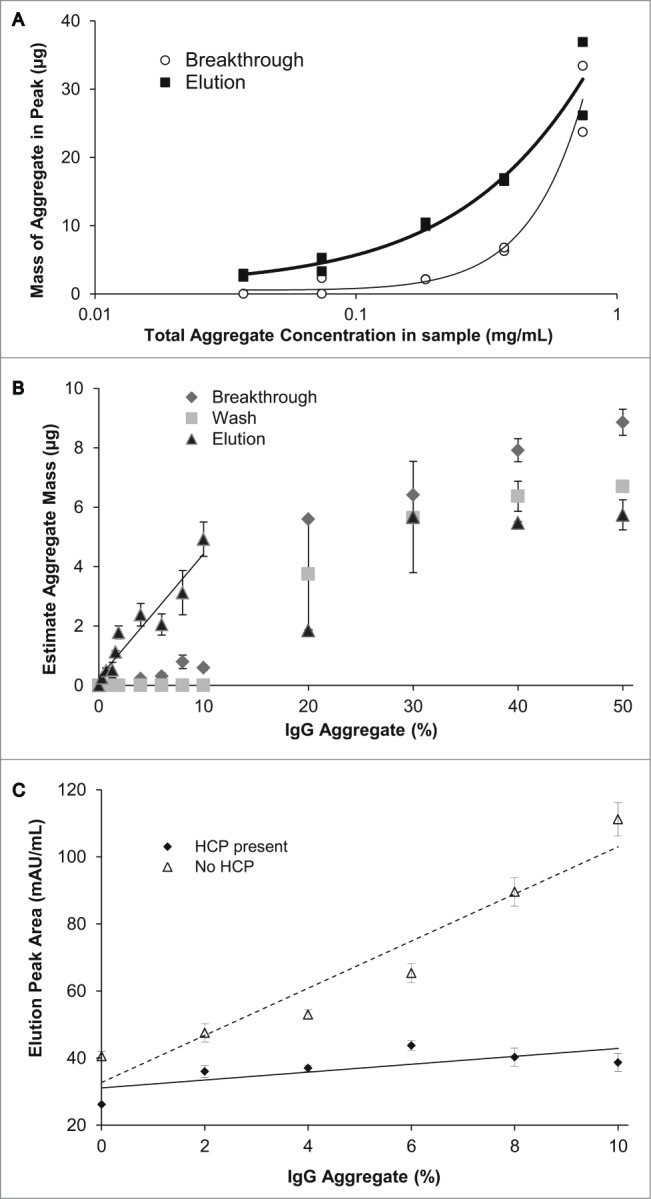
(**A**) Mass of aggregate present within the elution peak (black square) and breakthrough peak (open circle) against increasing concentration of aggregate within a given sample. (**B**) The estimated mass of aggregate using the NMCF-EVOH-SP method from the breakthrough, wash and elution peak fractions against increasing IgG aggregate percentage (0% to 50%). A linear regression line (R^2^ = 0.91) is shown for the elution data from 0% to 10% IgG aggregate. (**C**) Elution peak area against IgG samples with increasing percentage of aggregated IgG (0%, 2%, 4%, 6%, 8%, and 10%) in the absence (dashed line) or presence (solid line) of HCP Stock (**Fig. 1**). All tested samples had an IgG concentration of 1 mg/mL.

The NMCF-EVOH-SP was further tested with increasing concentrations of Stock B, using only a bind and elute method. Samples with an aggregate concentration of 0.74 mg/mL had more aggregate within the breakthrough, exceeding the amount eluted from NMCF-EVOH-SP (**Fig. 3A**). Thus, the NMCF-EVOH-SP can only effectively estimate aggregate concentration below this level. Increasing the level of aggregate above 10% in the purified antibody sample resulted in significant levels of aggregated material in both the unbound and wash fractions (**Fig. 3B**). As the aggregate concentration increased above 10%, too much of the aggregate was lost in the breakthrough and wash for a reliable estimate of aggregate to be made, thus limiting the range for reliable aggregate estimation to 10% or lower for the current 5 m length NMCF-EVOH-SP.

When the Protein A-purified IgG aggregate standards were diluted with the IgG depleted conditioned fermentation medium (CM) (unbound fraction from Protein A purification), the elution peak area was found to have a reduced correlation (R^2^ value of 0.54) to the aggregate level (**Fig. 3C**).

**Figure 4 (See previous page).**
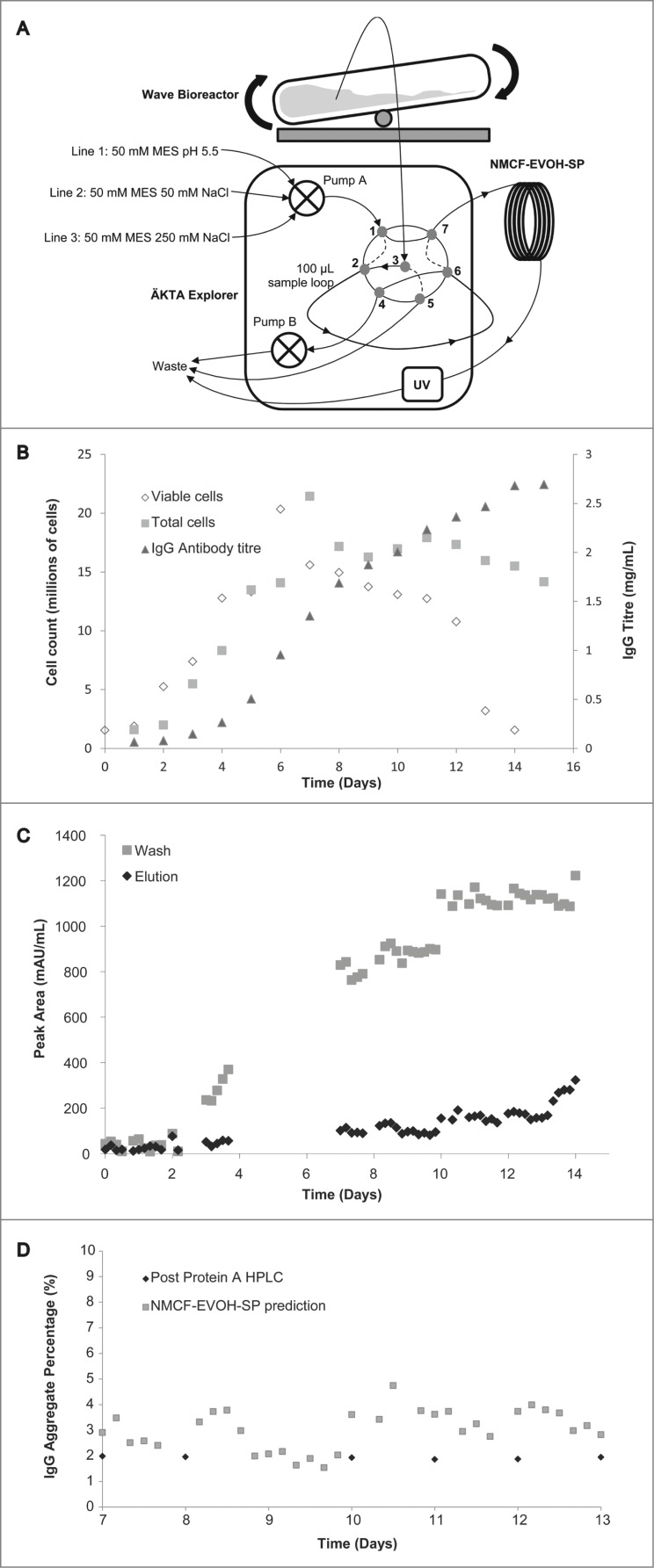
(**A**) Schematic representation of the at-line experimental setup. The WAVE Cellbag as well as the NMCF-EVOH-SP are connected via tubing to the numbered valves of the ÄTKA Explorer. The NMCF-EVOH-SP is also connected to the ÄTKA's UV detector for monitoring. Arrows indicate the direction of liquid flow. Within the valve, the solid lines indicate the flow path in the ‘load’ position, and the dashed lines indicate the flow path during ‘inject’. (**B**) Left Y-axis: total cell counts (gray square) and viable cell counts (white diamond) and right Y-axis: IgG titer (black triangle) against time in the bioreactor post-inoculation. (**C**) At-line bioreactor monitoring on samples collected every 4 hours using the NMCF-EVOH-SP method showing the wash peak area (gray square) and elution peak area (black diamond) against time in the bioreactor post-inoculation. A valve failure prevented collection of samples between days 4 and 7. (**D**) Aggregate percentage as predicted by at-line NMCF-EVOH-SP (gray square), compared to the standard Protein A / SEC HPLC method (black diamond) against time in the bioreactor post-inoculation.

The NMCF-EVOH-SP was directly attached to a WAVE bioreactor in order to establish a working model of at-line analysis (**Fig. 4A**). On day 6 of the fermentation, the viable cell density peaked at 20.3 × 10^6^ cells/mL with the antibody titer reaching 2.7 mg/mL on day 14 (**Fig. 4B**). The NMCF-EVOH-SP method was run every 4 hours throughout the culture process. During the course of fermentation, the elution peak area increased and thus predicted an increase in IgG aggregate, as determined by the NMCF-EVOH-SP method (**Fig. 4C**). However, by the standard technique of Protein A purification followed by SEC HPLC analysis, the aggregate level appeared to remain relatively constant (**Fig. 4D**); this result is discussed below.

Aggregates are a product variant, which makes them very difficult to remove and analyze because they share similar properties to the desired product. As aggregates are formed of several monomer proteins associated together, they have a greater net charge.^9^ Based on this property, NMCF-EVOH-SP was optimized to selectively isolate aggregates and provide a quick assay for determining the aggregate concentration within a given sample.

During IgG aggregate analysis with the NMCF-EVOH-SP method, the majority of aggregate is bound to the NMCF and only eluted with a relatively high salt wash (250 mM NaCl) (**Fig. 2A**). Based on evaluation of purified and non-purified samples containing monomeric as well as aggregated antibody, there is good correlation between aggregate level and elution peak area, at least up to 10% aggregate (**Fig. 3B**). The preferential binding of aggregate IgG over monomer is most likely due to the higher charge density exhibited by aggregated IgG molecules.^18^

The limit of the method to accurately measure aggregate levels above 10% has been attributed to saturation of the NMCF-EVOH-SP material. This can be clearly seen as a sharp increase in the unbound fraction at aggregate levels above 10% (**Fig. 3B**). The capacity of NMCF-EVOH-SP could be increased by using longer lengths of NMCF-EVOH-SP, although this would also increase the back-pressure, and therefore require a lower flow rate, increasing the sample processing time beyond the 20 minutes needed for the 5 m film or by reducing the amount of material loaded onto the NMCF-EVOH-SP.

Non-specific interaction of fermentation media components also appeared to compete with aggregated IgG, causing a depression in the response when compared with purified samples (**Fig. 3C**). This result could be exacerbated by the relatively modest concentration of IgG antibody in the mid-process samples tested (1 mg/mL), whereas IgG titers from modern fed-batch processes seen within industry can be substantially higher, particularly at the reactor harvest point where IgG values as high as 10 mg/mL have been reported.^19^

During the course of Chinese hamster ovary (CHO) cell culture, the at-line analysis data provided by the NMCF-EVOH-SP method revealed fluctuations in IgG aggregate levels between ∼2% and 5%. The traditional Protein A / SEC HPLC method suggested that endogenous levels of aggregation remained relatively constant at around 2% (**Fig. 4D**). Theoretically, the difference in these results can be explained by the NMCF-EVOH-SP method measuring *total* aggregated species whereas the Protein A / SEC HPLC method only quantified aggregated species that bind to Protein A in a filtered sample.^20^ Furthermore, it is possible for aggregate species to form or disassociate due to the purification conditions.^18^ However, without purification of the sample, SEC-HPLC would potentially give an overestimate of aggregate percentage because some HCP species have a similar retention time to aggregate proteins. Future work could incorporate an independent method of IgG aggregate analysis requiring minimal sample preparation to test this hypothesis. This could be achieved with analytical ultra-centrifugation, a method that does not alter the solvent conditions, nor require pre-treatment of the sample, creating a substantially lower risk of a non-representative result over SEC HPLC.^21^

In conclusion, NMCF-EVOH-SP can be used for the detection and quantitation of aggregated IgG in an at-line format with CHO fermentations. The critical advantage of this method is its ability to rapidly work in the presence of complex feed streams to generate near real time data without the need for sample pre-treatment. This capability could be very important to evaluate the effect of cell culture conditions and modulate parameters to minimize this common product-related impurity. The technique could also be used in other cell culture scenarios, such as during clone selection to screen for clones that produce higher aggregates. We envisage use of this type of at-line product quality testing to support the development of dynamic control strategies of both (fed-) batch and continuous fermentation strategies. Importantly, because the main chromatography component is made from an inexpensive material, NMCFs with different selective chemistries can be developed as disposable bioprocess technologies, offering the prospect of a range of quality attributes being tested at-line using this approach.

## Materials and Methods

### Materials

Poly(ethylene-vinyl alcohol) copolymer (EVOH) containing 32 mol% of ethylene was supplied by Eval-Europe (Cat. No. F101B). NaOH (Cat. No. S5881), cyanuric chloride (Cat. No. C95501), acetone (Cat. No. 34850), Na_2_HPO_4_ (Cat. No. S3264), 3-amino-1-propanesulfonic acid (Cat. No. A76109), 2-(*N*-morpholino)ethanesulfonic acid hydrate (Cat. No. M8250), Tris-HCl (Cat. No. T3253), sodium acetate (Cat. No. S8750), acetic acid (Cat. No. 695092), PBS tablets (Cat. No. P4417) and NaCl (Cat. No. S3014) were supplied by Sigma-Aldrich. MabSelect SuRe Protein A (Cat. No. 17-5438-01), Cation exchange Capto S (Cat. No. 17-5441-03), size exclusion resin HiLoad 16/600 Superdex 200 pg (Cat. No. 28-9893-35) and 22 liter WAVE Cellbag (Cat. No. CB0022L10–02) were supplied by GE Healthcare. Amicon 10 kDa centrifuge concentrators were supplied by Millipore, (Cat. No. UFC801024A) A MedImmune proprietary human IgG antibody with molecular weight of ∼150 kDa, and an isoelectric point above pH 7.0, produced from a MedImmune CHO cell clone, was used for all experiments.

### Production of NMCF-EVOH disc

Nineteen-capillary NMCFs were fabricated with a capillary diameter of 142 μm and a total width of 8 mm by melt extrusion of poly(ethylene-vinyl alcohol) copolymer at 200°C as described by Hallmark et al.^11^ Five meter lengths of NMCF-EVOH were spirally-wound into discs and the 2 exposed ends potted into ¼-inch HPLC connectors (using Upchurch components P-652, P-684, U-660X, U-662X and 1652) using slow-setting epoxy-resin (Araldite® Rapid).

### Surface modification of NMCF-EVOH disc with sulfonic acid chemistries

The internal surfaces of the microcapillaries in the NMCF-EVOH disc were functionalized for cation exchange chromatography using a procedure adapted from McCreath et al.^22^ and detailed in Darton et al.^12^ Briefly, the 5 m length of NMCF-EVOH was attached to an HPLC pump and placed in an ice bath. Thirty mL of ice-cold NaOH (1 M) was first recycled through the NMCF for 30 min in order to form alkoxide groups on the vinyl alcohol on the NMCF surface. Then, 20 mL ice-cold cyanuric chloride (50 mM) in acetone was recycled through the NMCF in an ice bath for 20 min, followed by a wash with 10 mL ice-cold MilliQ water for 10 min. This was followed by the addition of the cation exchange group using a 20 mL solution of 180 mM 3-amino-1-propanesulfonic acid, 100 mM Na_2_HPO_4_, pH 9.1. This solution was recycled at 1 mL/min through the NMCF-EVOH at 40°C for 17 hours. The temperature was increased to 60°C and recycling of the reagent was continued for a further for 5 hours. Twenty mL of MilliQ water was then passed through the NMCF for 20 minutes, followed by 20 mL of NaOH (0.4 M) for 20 minutes, and finally a wash with 20 mL MilliQ water for 20 minutes. The modified NMCF-EVOH-SP was then stored at 4°C in 20 mM Tris-HCl, pH 7.6.

### Preparation of immunoglobulin G aggregate samples

Three stock solutions of aggregated IgG antibody were created from pre-purified Protein A eluate antibody solutions (**Fig. 1**). The antibody was produced using a CHO cell line grown in serum-free medium, both proprietary to MedImmune, in 500 mL cultures with a seeding cell population of 0.3 × 10^6^ viable cells/mL, agitated at 120 rpm in a humidified incubator at 36.5°C and split every 3 to 4 d. Once a sufficient cell density was achieved, the cultures were clarified by filtration (1.2 µm, 0.45 µm and 0.22 µm membrane filter, PALL) before analysis.

The clarified harvest was loaded onto 20 cm bed height MabSelect SuRe Protein A columns, using PBS buffer (pH 7.2) for equilibration and wash, 50 mM sodium acetate (pH 3) for elution and 0.1 M acetic acid to recover the column. The eluate was used for subsequent aggregate stock generation.

The stock solution containing 0% aggregate, herein referred to as Stock A, was made by purification of the IgG isolated by Protein A chromatography using a Capto S cation exchange column, equilibrated and washed with 50 mM sodium acetate pH 5, and eluted in a gradient of 10 CV up to 300 mM NaCl, whereupon the eluate contained ∼0% aggregate.

The second stock solution of high aggregate IgG (˜10% aggregate), herein referred to as Stock B, was derived from a culture grown under nutrient restricted growth conditions (i.e., for 2 weeks without glucose addition to the culture media). This resulted in an aggregate level of ∼10% post-Protein A. Stocks A and B were blended to form a range of aggregate containing samples (0%–10%).

The third stock (Stock C) was made to replicate the highest levels of aggregate seen in industry from bispecific antibodies (>10%).^18^ This was achieved with SEC (using HiLoad 16/600 Superdex 200 pg) to isolate the aggregate from Stock B, creating Stock C (∼100% aggregate). The column was equilibrated for 1.5 CV, 20 mL sample injection, followed by 3 CV wash with 50 mM sodium acetate pH 6. Stock C was then blended with Stock A to create a range of aggregate samples ranging between 20% and 50% aggregate.

In order to determine the selectivity of NMCF-EVOH-SP for the analysis of aggregates in the presence of media components, a stock was prepared that contained only IgG-depleted CHO conditioned media (Stock HCP, **Fig. 1**). This was achieved by collecting the flowthrough from a Protein A column (MabSelect SuRe), which removed the IgG antibody from the culture fluid. The Protein A flowthrough (containing the host cell proteins and other cell and media components) was then spiked with the other stock solutions to a target antibody concentration of 1 mg/mL.

### IgG aggregate analysis by SEC HPLC

Aggregate levels were determined by with SEC HPLC using a TSKgel 3000 SW_XL_ column (7.8 mm × 30.0 cm, TOSOH Bioscience, Cat No. 08541) with a SW_XL_ guard column (6.0 mm × 4.0 cm; TOSOH Bioscience, Cat. No. 08543), connected to an Agilent 1100 HPLC system equipped with a 280 nm UV detector, running at 1 mL/min. Only an estimated mass could be calculated based upon the SEC HPLC peak area; a mass balance was not possible due to the loss of antibody during the use of centrifugal concentrators, and the limits upon the sample volume of SEC HPLC.

### IgG aggregate analysis with NMCF-EVOH-SP

To determine the ability of NMCF-EVOH-SP to bind and elute IgG antibody, samples were injected using a 100 μL injection loop of an ÄKTA Explorer (GE Healthcare). The flow rate throughout was 2 mL/min, with a total run time of 18 minutes. The UV absorbance was monitored at both 280 nm and 214 nm. NMCF equilibration was achieved by passing 4 CV, each of 1.54 mL, of 50 mM MES buffer, pH 5.5. The 100 µL sample loop was loaded with aggregate-containing sample and then passed through the NMCF-EVOH-SP, followed by a wash step to remove unbound protein (4 CV 50 mM MES, pH 5.5). The wash was conducted by passing MES buffer with salt within the ranges of 25 to 75 mM NaCl (8 CV). Subsequently, a step elution to remove any remaining protein using 50 mM MES buffer containing 250 mM NaCl was applied to the NMCF (4 CV). Fractions were taken throughout the process. Finally the NMCF-EVOH-SP was re-equilibrated as above. Collected fractions were used only to confirm the presence of aggregate species. These fractions were first concentrated from 3 mL to 200 µL using 10 kDa centrifuge concentrators (Millipore, Cat. No. UFC801024) and then analyzed by SEC HPLC (as described above).

### IgG aggregate analysis at-line with a bioreactor NMCF-EVOH-SP

NMCF-EVOH-SP capillaries have a diameter of 142 μm, which is sufficiently large to allow passage of eukaryotic cells (10–15 μm in diameter). Samples containing cells were directly loaded from a bioreactor through a check valve into a 100 μL sample loop attached to an ÄKTA Explorer using the B pump on the system in a custom setup to draw the sample through the injection valve into the sample loop (**Fig. 4A**). Drawing the sample avoids sheer damage to cells that potentially would have occurred if they had been passed through the pump. The sample once loaded was then injected onto the NMCF-EVOH-SP following the method described above.

The bioreactor, a customized 10 L working volume WAVE Cellbag was inoculated at an initial cell density of 1.3 × 10^6^ viable cells/mL in the initial 3 L. This was increased to a final volume of ∼6 L with four 750 mL nutrient feed additions during the 14 day production run. Each day cell counts were measured on an automated cell counter (Vicell, Beckman Coulter) and glucose was measured on a BioProfile FLEX analyzer (NOVA biomedical). Alkali and glucose solutions were supplemented into the culture to maintain pH and glucose respectively. Dissolved oxygen, temperature and mixing were controlled by the WAVEPOD™1 and 20/50EH rocking platform (GE Healthcare). Cell-free samples were frozen down each day in order to measure the total IgG titer using a MedImmune proprietary analytical Protein A method and the aggregate concentration using the SEC HPLC method as described above.
